# Supplementary material for: German funders’ data sharing policies—A qualitative interview study
Source: PLoS One. 2024 Feb 8;19(2):e0296956. doi: 10.1371/journal.pone.0296956 (PMC10852319; doi:10.1371/journal.pone.0296956)
Supplement: S2 Appendix — (DOCX) [file pone.0296956.s002.docx]

**Introduction to the expert interview:**

- Thank you very much for taking the time for the interview. Before the interview, you received some information about our project and the purpose of the interview, as well as form for informed consent. I would now like to start discussing the most important aspects, answer your questions and obtain your consent. Our research project is called „Carrots and Sticks? **Data** Sharing Policies for (German) Pu**blic** Research Funders. Ethical, Legal, Social and Behavioral Aspects (**Datablic**)“. It is funded by the German Ministry of Education and Research (**BMBF**)
- We investigate how public research funding agencies could and should design their funding policies, particularly their data sharing policies, in order to ensure that funded projects share their research data with the scientific community. In other words: how can data sharing policies best foster data sharing?
- We conduct expert interviews with representatives of organizations we found progressive in terms of their research funding policies in terms of data sharing. We are particularly interested in your personal experiences and perceptions in this area and want to gain insight into state-of-the-art data sharing practice. We would like to record and transcribe the interview, in order to perform a comparative content analysis later on. This serves as a basis for a legal and ethical evaluation of the findings.
- All audio and text material based on the interview will be treated confidentially and anonymously, of course. I.e., all personal data that can be used to draw conclusions about you, will be deleted or pseudonymised. This also applies, if we cite certain parts of the interview as part of a publication. All associates of the project are bound by confidentiality. Data won’t be stored in any data cloud, files will be password-protected.
- Please note that a revocation of your consent is possible at any time. You can chose to not answer questions, to pause or to end the interview at any time, without providing any reasons.
- Do you have any further questions about the procedure?
- I would now like to start the audio recording to obtain your verbal consent. Is that all right?

The recording of our conversation is now running after you agreed to this procedure. I will now read out a short text to ask for your consent.

- I, [name], hereby confirm that I have read the enclosed information and have been informed about the purpose of the study and the framework of the interview.
- I was given the opportunity to ask questions. All my questions were answered to my satisfaction.
- My participation in the study is voluntary and I have been given sufficient time to make this decision.
- I have received a copy of the Datablic project information document and the pre-interview consent form and have read them.
- I hereby consent to the Datablic Project procedure as previously described. I give my consent for the interview to be recorded and for the anonymised data obtained from it to be made available for the purely scientific purposes of the Datablic project.

Would you please both state your name and say whether you consent to these terms? Thank you very much.

| **Topic 1: Research organisations in the context of the Open Science landscape (10 minutes)** |  |
| --- | --- |
| 1. In the context of our research project, we are primarily concerned with the role of research funding organisations in the practical implementation and dissemination of data sharing, however, we have found that a considerable part of the actual expertise in this area is located at the level of the research data infrastructure (e.g., research institutes, universities, repositories, etc.).   - How would you describe the role of your own organisation in the context of the (open) research data landscape? - How did you come to develop this expertise and infrastructures? - Could you tell us about any reference points between your organisation and research funders?   2. Was there any form of influence or involvement or guidance from the government (e.g. BMBF) in these developments?   - Do you have to comply with any specific laws or regulations when it comes to sharing (managing) or supporting research data? - Do you perceive specific legal problems in your daily work with regard to research data and Open Science?   3. Data sharing and data management are very complex processes involving different stakeholders with different rights and needs. This points to the possible existence of ethical issues and problems.   - What ethical aspects and problems do you perceive regarding the sharing of research data? - How does your organisation deal with these? To what extent do you support or guide the handling of these? |  |
| **Topic 2: Experiences with Open Science and Data Management (10 Minutes)** | |
| 1. To the best of our knowledge, there are many different challenges regarding research data management in practice.  - Which problems do you perceive as particularly common or particularly serious for researchers? - How do you try to address or solve these problems? - How do you support researchers? 2.  1. Are you in personal exchange and discussion with...  - … researchers at your university? If yes, what feedback do you receive? - … other actors in the research data landscape? - … funders, project sponsors or other governmental/quasi-governmental actors?  1. How do you evaluate the general status quo of research data management at your institution, or beyond?  - Are you satisfied with the state of research data management and data sharing? - Do you see progress being made? - If you had the opportunity to change something, just like that, what would it be? | |
| **Topic 3: Legal and ethical challenges for research data management and sharing (20 minutes)** | |
| 1. How does your organisation manage intellectual property rights and copyright claims of its researchers in relation to research data?  - Who do you consider to be the owner of the research data within the research process? - Do you consider ownership of data to be contrary to or an obstacle to sharing research data in practice?  1. Do you think that an obligation to share research data, e.g., through funding guidelines, contradicts the scientific freedom of researchers? How much do you think mandatory funding guidelines influence the behaviour of funded researchers? What would you recommend? 2. Who is responsible and accountable for the quality and security of research data at different stages of the research and archiving process?  - Where do you store research data? Who pays for the storage? - Do you perceive any problems or conflicts here?  1. In the international research data landscape, Data Management Plans are an increasingly common phenomenon and are required by various funding bodies. DMPs require researchers to provide ethical and legal information about their research. (e.g., when it comes to considering the rights and needs of data donors/study participants like confidentiality, privacy).  - How do you perceive this development? - Do you also advise and support on such technical and legal requirements? - As a research organisation, do you have specific policies and/or procedures for personal/sensitive data?   5. Do you have a policy on access to research data? For example, do you have an Internal review Board/Data Access Committee? What problems do you perceive? | |
| **Topic 4: Monitoring, incentives and sanctions (5 minutes)** | |
| 1. Do you enter into a contractual relationship with funders once a project of a scientist from their organisations is funded? Are you expected to monitor funded researchers and their compliance with the funding guidelines? 2. A repeatedly expressed view is that sharing research data requires "carrots and sticks", both incentives and sanctions for deviant behaviour.  - What possible incentives for DS do you perceive? Which do you find good/bad? - What incentives does your organisation provide? - What is your own motivation in this process? Why do you contribute to it? - What do you expect from funding organisations? | |
